# Lead Poisoning and Insanity

**Published:** 1900-08-25

**Authors:** 


### LEAD POISONING AND INSANITY.

The injurious effects of lead upon many parts of the nervous system are so well recognised that it would have been strange if the more purely mental portions of the brain had not participated in its evil influence. Dr. Robert Jones, of the Claybury Asylum, read a paper at Ipswich on the occurrence of insanity among lead workers, in which he stated that out of the 1,950 male patients in residence at his asylum no less than 35 owed their mental disorder to lead poisoning. Among the occupations of the sufferers were those of house painters, glaziers, plumbers, gilders and workers in electro-plating, smelters of lead ores, and workers among pottery and china. The trades, however, which gave rise to the greatest dangers in this respect were the making of white and orange lead compounds, the dust of which contained the metal. The symptoms of lead encephalopathy observed in 19 patients at the Claybury Asylum were varied, among them being partial or total blindness, often of sudden or rapid onset, attacks of partial paralysis, convulsions and fits of an epileptic or hysterical nature, maniacal excitement, melancholic depression, attacks of delirium and mental confusion, and apathy and dementia. Some of the cases showed symptoms very like those of general paralysis. Headache and loss of memory were met with, and also hallucinations of sight and hearing, with delusions of persecutions. The epileptic fits which occurred in seven out of the 19 cases seemed to be of toxic origin, like those of uræmia or eclampsia. The majority of the cases benefited under treatment.

